# Differential Regulation of Drought Responses in Two *Phaseolus vulgaris* Genotypes

**DOI:** 10.3390/plants9121815

**Published:** 2020-12-21

**Authors:** Cristina María López, Manuel Pineda, Josefa M. Alamillo

**Affiliations:** Departamento de Botánica, Ecología y Fisiología Vegetal, Grupo de Fisiología Molecular y Biotecnología de Plantas, Campus de Excelencia Internacional Agroalimentario, CEIA3, Campus de Rabanales, Edif. Severo Ochoa, Universidad de Córdoba, 14071 Córdoba, Spain; b22lovac@uco.es (C.M.L.); bb1piprm@uco.es (M.P.)

**Keywords:** ABA-responses, landraces, common bean, resistance, senescence, WRKY transcription factors

## Abstract

Drought is probably the most harmful stress affecting common bean crops. Domestication, worldwide spread and local farming practices has entailed the development of a wide variety of common bean genotypes with different degrees of resistance to water stress. In this work, physiological and molecular responses to water stress have been compared in two common bean accessions, PHA-0683 and PMB-0220, previously identified as highly and moderately resistant to water stress, respectively. Our hypothesis was that only quantitative differences in the expression patterns of key genes should be found if molecular mechanisms regulating drought resistance are similar in the two accessions. However, results presented here indicate that the resistance to drought in PMB-0220 and PHA-0683 common bean accessions is regulated by different molecular mechanisms. Differential regulation of ABA synthesis and ABA signaling related genes among the two genotypes, and the control of the drought-induced senescence have a relevant contribution to the higher resistance level of PHA-0683 accession. Our results also suggest that expression patterns of key senescence-related transcription factors could be considered in the screening for drought resistance in common bean germplasm collections.

## 1. Introduction

Drought is one of the most harmful abiotic stresses affecting yield of common bean (*Phaseolus vulgaris*) crops, with a great economic and dietary importance, especially in developing countries. Moreover, current climate change and its associated increase of drought episodes are expected to worsen the negative effect of water stress in crops productivity worldwide [1,2,3,4]. Like other legumes, common bean can use atmospheric nitrogen (N_2)_ through the symbiotic fixation process [5], thus allowing a reduced use of fertilizers, promoting a more sustainable agriculture. Typically, the uredic legumes such as common bean (*P. vulgaris*) and soybean (*Glycine max*) transport the symbiotically fixed nitrogen in the form of the ureides, allantoin and allantoate, whereas most temperate legumes transport their N in the form of amides [6,7]. Symbiotic nitrogen fixation is rapidly inhibited under conditions of water stress [8], and the ureidic legumes are in general more susceptible than the amidic ones. Several studies suggested that accumulation of ureides during water stress could be related with the inhibition of symbiotic fixation in ureidic legumes [9]. In contrast, subsequent studies have shown that nodule activity in plants subjected to drought is inhibited earlier than the accumulation of ureides in these plants [10,11,12]. Up to now, there is no a clear relationship between ureides concentration and inhibition of symbiotic fixation during drought, but, on the contrary, also a possible protective role of ureides has been suggested in several plants, probably through their effects regulating abscisic acid (ABA) and jasmonate (JA) levels [11,12,13,14].

The phytohormone abscisic acid (ABA) is a key regulator of the plant response to abiotic stressors, but especially to osmotic and water deficit stress. The biosynthesis of this hormone is activated in response to dehydration, through the oxidative cleavage of carotenoids. In contrast, its degradation is induced by rehydration [15], modulating the ABA content according to the environmental conditions. The first steps of its synthesis, from β-carotene to xanthoxin, occur in the plastids, while the final steps take place in the cytosol [16,17]. ABA regulates a plethora of physiological processes, as stomatal closure, growth, as well as dormancy and germination of seeds [18,19,20]. ABA-mediated responses are also involved in the regulation of other plant developmental processes such as leaf senescence and abscission [21]. In response to water deficit, ABA levels rise and initiates the ABA-signaling of these stress responses [22]. ABA is recognized inside the cells mainly by a family of ABA receptors named Pyrabactin Resistance (PYR)/ PYL(PYR-Like), or Regulatory Component of ABA Receptor (RCAR) [23,24]. In the presence of ABA, the ABA receptors PYR/PYL/RCAR bind to, and inactivate type 2C (PP2Cs) Ser/Thr protein phosphatases [23,24,25]. Inactivation of PP2Cs allows the activation of SnRK2 kinases (Snf1-related protein kinase class 2), which subsequently phosphorylate ABI5/ABFs transcription factors (ABA-Insensitive5/ABA-responsive element binding factors) which in turn activate the downstream effectors of the stress response [26,27].

Increases in plant resistance to water deficit have been associated to promotion of leaf senescence, in part induced by changes in ABA levels under stress conditions [21]. The outcome of leaf senescence is the mobilization of nutrients from senescent to younger tissues, as well as the death of cells with the consequent foliar abscission [28], which, in turn, reduces leaf surface and transpiration rates. It has been suggested that ABA-induced senescence was promoted by the biosynthesis of ethylene [29]. However, more recent studies showed that, in *Arabidopsis thaliana,* ABA-induced foliar senescence is mediated by an ethylene independent pathway, through direct inhibition of PP2Cs, activation of SnRK2s and activation of the downstream signaling pathway [30]. Among the downstream genes involved in activation of senescence and ABA-mediated responses to drought are some of the WRKY transcription factors (TF) [31,32]. WRKY proteins constitute large families that have been classified into three groups depending on its characteristic WRKY domains and zinc finger motifs [33]. The number of WRKY proteins varies in each species, with 74 and 88 *WRKY* genes identified in *Arabidopsis* and *P. vulgaris,* respectively [34,35], while up to 197 WRKY proteins have been found in *G. max* (soybean) [36]. Although WRKY TFs have been extensively studied in a wide variety of plants, their specific roles in the regulation of drought resistance and senescence in legumes is still largely unknown.

Although common bean is susceptible to drought conditions, finding varieties better adapted to environmental stresses will have a major impact on crop yields and human nutrition, particularly in developing countries [37]. The domestication processes and local farming practices have led to the development of a wide variety of common bean genotypes with different degrees of resistance to drought stress [3,38]. Characterization of a high number of accessions from the common bean Spanish collection revealed superior performance of several accessions under water scarcity conditions. From these the PHA-0683 landrace appeared as a high yield resistant genotype, whereas the commercial breeding line Great Northern PMB-0220 ranked as a slightly less drought-resistant line [39]. 

Although resistance to drought has been evaluated in many collections of bean germplasm, obtaining varieties resistant to water stress while keeping good productivity is a complex task, because resistance to drought is a trait largely affected by environmental interactions with additive and quantitative effects [40,41]. Molecular analyses, boosted by the recent release of common bean genomes, are important tools to understand how drought resistance is achieved in these crops. Genome-wide drought responsive genes have been identified by transcriptomic analysis in several legume crops [42,43,44], including common bean [45,46]. 

Previous studies from our group showed that PHA-0683 landrace, besides showing a high yield performance, was also able to maintain good rates of N2 fixation and did not accumulate ureides in response to the drought stress [12]. On the other hand, the PMB-0220 line showed a good level of resistance to drought, although lower than that of PHA-0683 [12,39,40]. In addition, a genome wide transcriptomic analysis of differentially expressed genes under drought conditions in the resistant common bean PHA-0683 landrace, identified changes in ABA signaling-related genes and several key transcription factors known to regulate stress and developmental responses [47]. 

The hypothesis of the actual work was that the different degree in resistance in the two genotypes studied here should be caused by differential regulation at the molecular level. Therefore, only quantitative differences will be expected, if the higher level of resistance in PHA-0683 and the lower in PMB-0220 are governed by similar regulatory mechanism. In contrast, qualitative differences in the expression patterns of key factors would reveal different mechanisms of resistance to stress in these two genotypes. Therefore, the main aim of this work was the comparison of physiological and molecular responses to drought in the common bean PHA-0683 and PMB-0220 accessions.

## 2. Results

To determine if the different degrees of resistance to water stress in two common bean genotypes are indeed mediated by differential molecular responses, the highly tolerant PHA-0683 landrace and the PMB-0220 breeding line [12,39] were subjected to 10 days of water deficit and the responses were compared to those of the well-irrigated, control plants, from each genotype.

### 2.1. Physiological Effects of Drought in Two Common Bean Drought Tolerant Plants

Drought effects on the relative water content of soil and leaves and in the plant biomass were compared between the control plants and the plants subjected to 10 days of water deficit on the PHA-0683 landrace and the PMB-0220 breeding line. 

**Figure 1:**
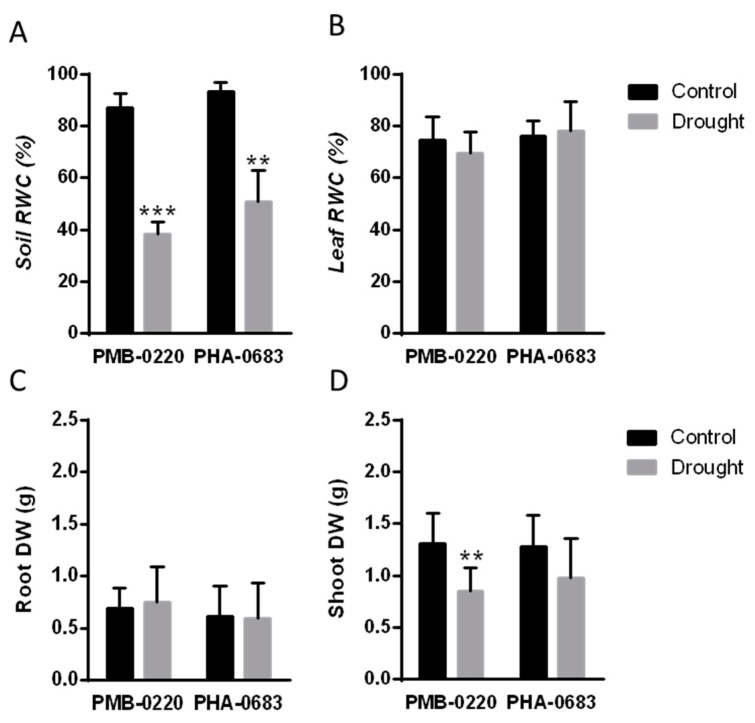
Physiological parameters of control and 10 days water deficit stressed plants of common bean PMB-0220 breeding line and PHA-0683 landrace, grown under symbiotic nitrogen fixation conditions for 28 days before the stress treatment. Relative water content of (**A**) soil and (**B**) leaf, and biomass of (**C**) roots and (**D**) shoots. Data are means of three independent experiments and asterisks indicate statistically significant differences (** *p* < 0.005) and (*** *p* < 0.0005).

The results in Figure 1A show that the soil moisture (relative soil water content) was above 80% of soil water capacity (field capacity) in the control group of both cultivars, whereas, after 10 days of drought treatment, soil water content decreased up to 40% in the PMB-0220 line and up to 50% SWC in the PHA-0683, thus suggesting that PHA-0683 plants used less water from soil than PMB-0220 ones. However, despite the shortage in available soil water content, there were no significant differences in the relative leaf water content (LWC) between control and drought conditions in any of the two cultivars (Figure 1B). Nevertheless, while PHA-0683 did not show any reduction in leaf water content after the water stress, a slight decrease in LWC was found in leaves of PMB-0220, agreeing with the degree of drought resistance in these plants. 

Determination of the effect of water deprivation in the biomass of roots and shoots showed that, although there were no significant differences in the total roots biomass between control and drought conditions in any accession, a slight increase in the biomass of roots, with respect to the control conditions, was observed in PMB-0220 plants (Figure 1C). In contrast, a significant reduction in shoot biomass was found in the water-stressed PMB-220 plants, compared to their control condition (Figure 1D). On the contrary, there were no significant differences between control and drought conditions in the aboveground biomass in PHA-0683 plants (Figure 1D). Moreover, under control conditions, similar biomass was found in the roots and shoots of the two genotypes, indicating that the moderate differences in water use and in shoot biomass were not caused by phenological or developmental differences of the plants. 

### 2.2. Changes in Total Chlorophyll Contents in Response to Drought

**Figure 2:**
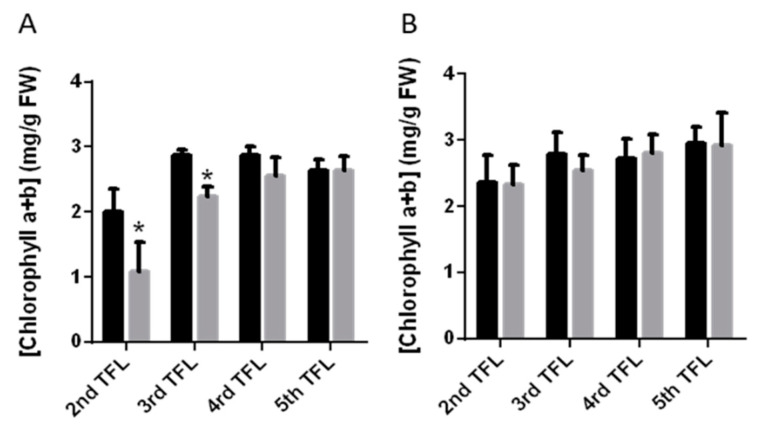
Concentration of chlorophylls in the 2nd, 3rd, 4th and 5th trifoliate leaves of *P. vulgaris* PMB-0220 (**A**) and PHA-0683 (**B**) plants under control conditions and after 10 days of drought. Data were means of three independent repetitions, and 3 plants were used per sample in each replicate. Asterisks indicate statistically significant differences (* *p* < 0.05).

Drought stress is known to trigger senescence symptoms leading to a reduction of chlorophyll contents in the leaves of stressed plants. To determine whether there where changes in the chlorophylls levels in response to the stress in the PMB-0220 and PMB-0683 genotypes, contents of both, chlorophylls a and b, were measured in all the developed leaves present in plants submitted to 10 days of drought treatment and in control, well-irrigated plants (Table 1). The results, summarized in the Figure 2A, showed that the oldest leaves in the drought stressed PMB-0220 plants had lower chlorophyll content than control ones. Moreover, although chlorophylls a and b were reduced in the second trifoliate leaves of PMB-0220 after 10 days of water stress, the reduction was higher in chlorophyll a than chlorophyll b (Table 1). Instead, the leaves of PHA-0683 drought stressed plants did not show any significant reduction in chlorophylls content with respect to its control conditions (Figure 2B). On the contrary, chlorophyll levels appeared to be slightly higher in the youngest leaves of the water-stressed PHA-0683 plants, probably reflecting the slight reduction of the area of the youngest leaves, that accounts also for the moderate decrease in shoot biomass in these plants.

### 2.3. Analysis of Genes Expression Related to ABA-Mediated Response

**Figure 3:**
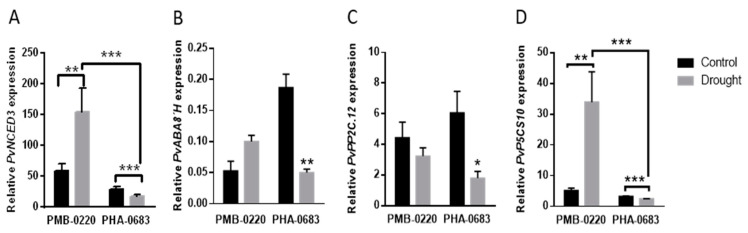
Relative expression of abscisic acid (ABA)-related genes in leaves from control and 10 days-drought-stressed plants of PMB-0220 and PHA-0683 lines, grown under symbiotic nitrogen fixation conditions. Relative expression of (**A**) *PvNCED3*; (**B**) *PvABA8’H*; (**C**) *PvPP2C.12* and (**D**) *PvP5CS10* in response to drought. Data were means of three independent samples and asterisks indicate statistically significant differences (* *p* < 0.05) (** *p* < 0.005) and (*** *p* < 0.0005).

In a previous study, several genes of the core ABA signaling were found to change in response to drought in the highly resistant PHA-0683 landrace [47]. To elucidate whether similar ABA-mediated responses take place in the PMB-0220 line, levels of relative expression of key genes for ABA synthesis (9-Cisepoxycarotenoid Dioxygenase, NCED), degradation ((+)-Abscisic acid 8’-Hydroxylase, ABA 8’H), ABA signaling (type 2C protein phosphatases, PP2Cs) and downstream responsive genes, as the proline synthesis (∆1-Pyrroline–5-Carboxylate Synthase, P5CS), were measured in samples of the two genotypes. Expression patterns of these ABA-related genes were compared in leaf tissues from control and from plants subjected to 10 days of drought from each genotype (Figure 3A–D). As shown in Figure 3A–D, the relative expression of *PvNCED3*, *PvABA8’H* and *PvP5CS10*, coding for ABA synthesis, ABA degradation and ABA-responsive genes, respectively, was significantly induced by drought in the PMB-0220 line. In contrast, the expression of these genes was repressed by water deprivation in PHA-0683. On the other hand, *PvPP2C.12*, coding for a phosphatase 2C involved in repression of ABA-responses [48], showed a reduction in its levels under drought conditions in both cultivars (Figure 3C), although *PvPP2C.12* gene expression was only highly repressed under drought conditions in PHA-0683, with only a slight, no significant decrease of expression levels in PMB-0220 samples (Figure 3C). 

### 2.4. Analysis of Genes Expression Related to Senescence in Response to Drought

**Figure 4:**
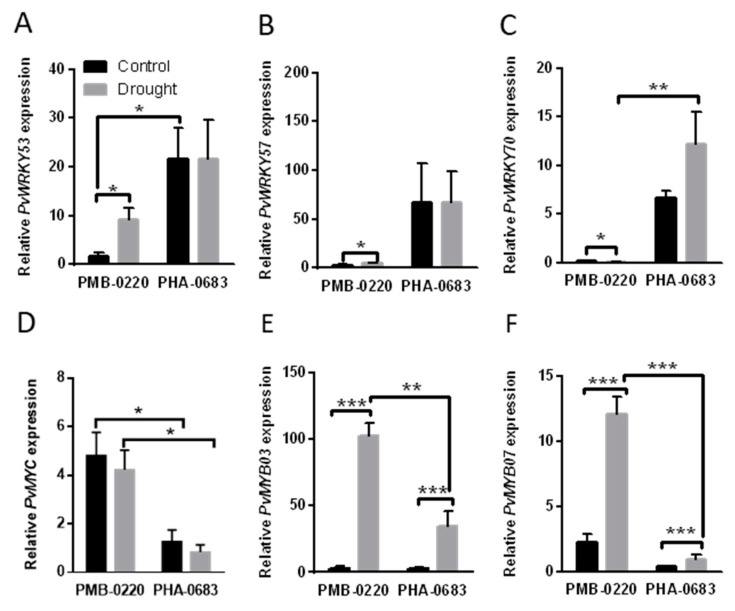
Relative expression of key transcription factors related with senescence and drought responses in leaves from control and 10 days-drought-stressed PMB-0220 and PHA-0683 plants, grown under symbiotic nitrogen fixation conditions. Relative expression of (**A**) *PvWRKY53*; (**B**) *PvWRKY57*; (**C**) *PvWRKY70*; (**D**) *PvMYC*; (**E**) *PvMYB03*; and (**F**) *PvMYB07* in response to drought. Data are means of three independent experiments and asterisks indicate statistically significant differences (* *p* < 0.05) (** *p* < 0.005) and (*** *p* < 0.0005).

To investigate if the differences in the ABA-related genes among the two genotypes could also be observed in transcription factors involved in key processes affecting drought resistance in the PMB -0220 and PHA-0683 lines, the expression of several genes related to the activation or inhibition of senescence, and whose expression could be mediated by ABA levels, was measured in leaf samples from control and drought stressed plants from the two genotypes. The results in the Figure 4A–C show that the drought-mediated changes in the expression of three genes encoding WRKY TF exhibited significant differences between the PMB-0220 and PHA-0683 plants in response to the stress. Expression of *PvWRKY53* gene, directly related with induction of senescence [49,50], showed a significant induction after 10 days of drought with respect to its well-irrigated control in the PMB-0220 line (Figure 4A). On the contrary, there were no significant differences in expression of PvWRKY53 between the control and stressed leaf tissues in the PHA-0683 plants (Figure 4A). However, *PvWRKY53* showed a higher expression level under control conditions in PHA-0683 than in PMB-0220. Moreover, the expression patterns of *PvWRKY57*, also related to induction of senescence and drought resistance [51], were similar to these of *PvWRKY53* in response to drought. *PvWRKY57* was significantly overexpressed at 10 days of water stress compared to the control conditions in the PMB-0220 cultivar, while in PHA-0683 there were no significant differences between control and water stressed samples, although, in this landrace, *PvWRKY57* was overexpressed with respect PMB-0220 (Figure 4B). In addition, the *PvWRKY70* gene, which has been related with inhibition of senescence [52], showed an opposite expression pattern in one cultivar or the other during drought conditions. *PvWRKY70* was significantly downregulated under drought conditions in PMB-0220, whereas it showed an induction of its expression at 10 days of water stress in leaves from PHA-0683 plants. Finally, the results presented in Figure 4D–F show that the expression of genes coding for MYC and MYB transcription factors involved in the responses to abiotic stress was also different among the two common bean genotypes. *PvMYC* was slightly downregulated under drought conditions in both cultivars, but its expression was significantly higher in the control and drought conditions in PMB-0220 than in PHA-0683. In contrast, two *MYB* genes, *PvMYB03* and *PvMYB07*, showed a significant induction of their expression after 10 days of drought in each genotype, although the level of induction was also higher in the common bean PMB-0220 than in the PHA-0683. 

## 3. Discussion

The search for drought resistant common bean varieties has increased due to the worsening of drought episodes by the current climate change conditions [37]. Most research until now has focused on the search for the most productive genotypes, based on the physiological and agronomical characterization of cultivars or landraces that evolved under such unfavorable conditions, or that have been obtained in breeding programs [39,40]. These studies have revealed that there are large phenotypic differences in the drought tolerance in common bean genotypes [3]. However, despite the large effort posed in the search for drought-resistant common bean lines, studies aimed to dissect the molecular mechanism that govern these phenotypic differences are scarce till now. In this work, molecular and physiological responses of two common bean genotypes have been compared. From these, landrace PHA-0683 ranked as a highly drought-resistant genotype, whereas PMB-0220 was a commercial high yielding line, that ranked as moderately resistant to drought conditions [39]. In a recent genome wide transcriptomic analysis in leaf tissue of PHA-0683 landrace, differential expression of key genes related with ABA-mediated responses and the regulation of senescence by drought was observed [47]. 

The results in physiological parameters shown in Figure 1 revealed that there were no significant differences in the leaf relative water content and biomass accumulation between the control and drought-stressed plants of PHA-0683 landrace, with only a slight reduction in the shoot biomass after 10 days of water stress (Figure 1D), which correlated to a discrete reduction in the number of pods and seeds caused by water deficit in field experiments [39]. However, common bean PMB-0220 breeding line showed a significant decrease in shoot biomass and a slight increase in root biomass under drought conditions against control conditions (Figure 1C,D). This slight root growth is a common response related to the need to reach soil layers with higher moisture levels [53]. The decrease in aerial dry matter in both cultivars also corresponded to a general slow-down of cell expansion, to reduce the gas exchange surface and the loss of water in the leaves in response to water scarcity. Moreover, the smaller effects observed in the drought-stressed PHA-683 plants agrees with the higher level of drought resistance reported for this landrace in comparison with the PMB-220 breeding line. Noteworthy, most of the physiological and phenological parameters related to growth habits, days to flowering, shoot and root biomass are similar in the two common bean genotypes [39]. Therefore, the small increase in root and decrease in shoot biomass in PMB-0220, but not in PHA-0683, should be explained as a differential response to drought, in turn, caused by metabolic and molecular changes in the two lines. Moreover, the higher relative soil water content after the water deficit treatment in the PHA-0683 landrace strongly suggests that this genotype is better adapted to restrict water loss, probably through a faster ABA-mediated stomatal closure [20,54]. 

In a previous study, the transcriptome changes in response to drought showed down-regulation of genes coding for proteins that are repressors of ABA signaling, as the PP2Cs, and of ABA degradation, as ABA 8′ hydroxylase, suggesting that ABA-mediated responses were activated in PHA-0683 landrace. [47]. In this work we have compared the expression levels of genes coding for key enzymes in ABA synthesis, as the 9-cis-epoxycarotenoid dioxygenase (NCED) [55], signaling as phosphatase PP2C, degradation as ABA 8′ hydroxylase, and ABA-responsible genes, like the one coding P5CS, involved in the synthesis of proline. 

Abscisic acid is synthesized de novo in response to dehydration, through transcriptional regulation of ABA biosynthetic genes [56]. Several studies have shown that NCED was the key enzyme involved in ABA biosynthesis [57,58]. Nine *NCED* genes have been identified in *Arabidopsis thaliana,* of which *NCED3* (*AtNCED3*) controlled the level of endogenous ABA under drought stress conditions [56]. In *P. vulgaris* there are three *NCED*-related genes, from which *Phvul.005G051600* showed the highest homology to *NCED3* from *Arabidopsis* and was the one chosen in this analysis.

In PMB-0220 plants, *PvNCED3* showed a significantly higher level of expression in drought-stressed than in control leaves (Figure 3A), suggesting induction of the synthesis of ABA during water deficit conditions. In contrast, the expression level of *PvNCED3* was lower under drought conditions with respect to the control in PHA-0683 landrace, and it was also lower than in water-stressed samples from the PMB-0220 genotype (Figure 3A). In contrast, the *PvABA8’H* and *PvPP2C.12* genes were down-regulated by water stress in PHA-0683, with respect to control conditions, while in PMB-0220, these genes were either up-regulated, or remained unchanged in response to stress (Figure 3B,C). *PvPP2C.12* gene encodes a PP2C phosphatase recognized as a negative regulator of ABA-mediated response [59], and *PvABA8’H* encodes the ABA-8’-hydroxylase protein that catalyzes the key oxidation step in the ABA catabolism pathway [60,61]. Therefore, expression patterns of these ABA-related genes strongly suggest different regulatory mechanism in the two cultivars. Thus, while water stress seems to promote the ABA biosynthesis in PMB-0220 cultivar, it reinforces the ABA-mediated responses in the PHA-0683 landrace by repressing negative regulators and inhibiting its degradation. Nevertheless, although 10 days of treatment were chosen because PHA-0683 did not show any appreciable symptoms of stress at earlier times [12], we cannot exclude that different kinetics could be responsible for these results; thus, further analysis should be done to determine whether ABA synthesis could be induced earlier in these plants. 

Apart from the ABA-mediated regulation of stomatal conductance, triggering stomatal closure, ABA also promotes the synthesis of compatible metabolites such as proline, which acts as active compounds to allow osmotic adjustment during water stress [62]. Increases in proline concentration is a widespread response to drought, cold, and salt stress, often regarded as a basic strategy for the protection and survival of plants under abiotic stress. Proline accumulation is mediated by the induction of the *P5CS* gene, encoding a Δ1-pyrroline-5-carboxylate synthase, which catalyzes the first and rate-limiting step in the synthesis of this amino acid. 

In most plants, there are two isoforms of P5CS that differ in their temporal and spatial expression patterns [63,64,65]. In common bean, there are four genes coding P5CS proteins, from which *PvP5CS10 (Phvul.010G015400)* showed the highest homology to P5CS1, responsible for the accumulation of proline under drought stress in *Arabidopsis.* The relative expression of *PvP5CS10* increased significantly after 10 days of drought in PMB-0220 (Figure 3D), suggesting that synthesis of proline might contribute to the osmotic adjustment in these plants that showed no significant differences in the leaf RWC under water stress with respect to the control (Figure 1B). In contrast, the proline synthesis gene, *PvP5CS10,* was repressed compared to control conditions in the PHA-0683 plants, despite that RWC was also maintained in leaves subjected to 10 days of drought in these plants. Interestingly, genes coding for glycine-betaine were found as induced by water stress in the transcriptome analysis of this landrace [47], suggesting different osmotic adjustment mechanisms in the two genotypes. Moreover, a recent study demonstrated that concentration of proline does strongly correlate with the susceptibility to drought in common bean landraces and accessions [66], thus also agreeing with the lower resistance of the commercial cultivar PMB-0220 compared to the PHA-0683 landrace.

Besides acting as a compatible osmolyte, proline produced under stressful conditions also acts as a free radical scavenger and an activator of ROS detoxification pathways. Moreover, the accumulation of proline can function as a signaling molecule, inducing the expression of stress-responsive genes, including those encoding enzymes that scavenge ROS [67]. Accordingly, preliminary results from our group indicate that induction of some antioxidant activities, like superoxide dismutase (SOD) and catalase, were higher in PMB-0220 plants than in the PHA-0683 ones (Supplementary Figure S1)

In addition to osmotic regulation, acceleration of leaf senescence is a common response of many plants to adapt to adverse conditions [30]. Leaf senescence is a developmental process regulated via changes in hormonal balance, especially associated with the decrease in cytokinin (CKs) and increase in ABA contents, although levels of jasmonic acid (JA), ethylene, and salicylic acid are also important [68,69]. These changes in hormonal balance are transduced by transcription factors including several members of the WRKY, NAC, MYC, and MYB TF families. We found that the expression of *PvWRKY53,* a key gene related to the induction of senescence in *Arabidopsis* [49], had a significant induction in leaves of PMB-0220 plants subjected to 10 days of water stress, while there were no significant differences among drought and control samples from PHA-0683 plants (Figure 4A). On the contrary, *PvWRKY70*, which is considered as a senescence repressor gene in *Arabidopsis* [50], was significantly upregulated in the foliar tissue of plants subjected to drought in PHA-0683 landrace (Figure 4C), confirming the results from the transcriptome study of these plants [47]. Conversely, *PvWRKY70* gene was downregulated in leaves from the PMB-0220 line subjected to drought. Besides being involved in senescence regulation, some genes coding for WRKY TFs are also directly related to the tolerance to water stress. *PvWRKY53* and *PvWRKY57* might be part of this group, as reported for their closest homologs in soybean (*GmWRKY54*) and *Arabidopsis* (*AtWRKY57*) [52,70]. The expression patterns of these TF, shown in Figure 4, strongly agree with the high tolerance of the common bean PHA-0683 landrace, where, in addition to the *PvWRKY70* inhibition of foliar (Figure 4C)*,* high levels of *PvWRKY53* and *PvWRKY57,* found already under control conditions, may promote the protection against water stress. Moreover, the expression of *WRKY* genes was always higher in samples from PHA-0683 than in the those from the PMB-0220 cultivar (Figure 4A,B), thus confirming the differential behavior of these regulatory genes in the two genotypes. 

Furthermore, MYB- and MYC-type transcription factors have been related with ABA and jasmonate-mediated responses that regulate tolerance to abiotic stresses as drought [51,71]. MYB transcription factors have also been associated with the regulation of senescence [72]. In this work, we investigated the expression of two MYB and one MYC encoding genes, whose expression was previously found to change in response to drought in PHA-0683 landrace [47]. Two genes that code for MYB TFs (*PvMYB03* and *PvMYB07)* were overexpressed in PMB-0220 and PHA-0683 after 10 days of drought (Figure 4E,F), although the induction was higher in PMB-0220 than in the PHA-0683 genotype, agreeing with the induction of ABA synthesis genes in PMB-0220 plants. In contrast, the MYC-coding gene showed no significant expression changes in any of the two lines, but, again, its expression levels were higher in the leaves from PMB-0220 than in those from PHA-0683 plants.

Analysis of the promoter regions of *PvWRKY53, PvWRKY57,* and *PvWRKY70* genes revealed that they contain several *cis*-acting elements related to the binding of MYC and MYB TF. Moreover, the promoters of these *WRKY* genes also contain other stress-related *cis*-acting elements, such as ABRE (ABA responsive element), TCA-element (salicylic acid responsive element), CGTCA/TGACG-motif (MeJa responsive element), P-box (gibberellin-responsive element), and TGA-element (auxin-responsive element) (Supplementary Table S1). The presence of several abiotic stress-related motives in the *WRKY* genes suggests that, indeed, they would play a prominent role in drought resistance in the two common bean cultivars. On the other hand, WRKY TFs regulate the expression of downstream genes involved in general features of senescence, as remobilization processes and induction of antioxidant enzymes. In fact, direct interaction of WRKY53 with the promoter of catalase genes has been reported [73]. Accordingly, we found induction of catalase activity only in drought-stressed leaves from PMB-0220, agreeing with the induction of *PvWRKY53* expression in these plants, but not in the PHA-0683 ones. 

Chlorophyll degradation is among the early symptoms of leaf senescence [74,75]. Determination of chlorophyll concentration in the two common bean genotypes revealed that chlorophyll degradation occurred earlier in the PMB-0220 than in the PHA-0683 plants submitted to water stress (Table 1; Figure 2). These results coincided with the more pronounced decrease in the aerial biomass under water stress in PMB-0220 plants and with the expression patterns of *PvWRKY53* and *PvWRKY70,* found in these two genotypes. 

Regulation of senescence under drought conditions may have both beneficial and detrimental effects and there are studies showing that the delay of foliar senescence is related to resistance to water deficit [21]. Contrary, there are many other reports highlighting that the increase in ABA levels directly promotes senescence, with the consequent remobilization of nutrients from senescing organs to the youngest tissues [30,76,77]. Inhibition of leaf senescence in PHA-0683 plants under drought is also consistent with a lower induction of *NCED* gene controlling ABA synthesis, compared to the results in PMB-0220 accession (Figure 3A). This also agrees with previously reported results showing that PHA-0683 plants are able to maintain symbiotic nitrogen fixation, reducing the need for nutrient remobilization [12]. In contrast, the promotion of senescence in the oldest leaves of PMB-0220 plants might help to acquire the required nitrogen to nourish young tissues under the water stress, thus also contributing to the drought resistance of these plants. Although further data are required to ascertain whether control of senescence is a unique adaptation of PHA-0683 landrace, or, instead, it could have an important role in the resistance of other common bean genotypes. Moreover, our results highlight the need to implement more broad-range molecular analysis, including whole genome sequencing and transcript abundance under environmentally stressful conditions in different bean genotypes, that will help to dissect drought resistance regulation in this important crop. 

In summary, we show that the drought tolerance in PMB-0220 and PHA-0683 common bean accessions is regulated by different molecular mechanisms and that control of the drought-induced senescence seems to be relevant for the better performance of PHA-0683 accession under drought conditions. Our results also suggest that expression patterns of key TF as *WRKY53* and *WRKY70,* or the proline synthesis *P5CS,* could be used for easy screening of drought resistance/susceptibility in common bean germplasm collections.

## 4. Materials and Methods

### 4.1. Growth Conditions and Plant Material

In this study, *P. vulgaris* highly drought resistant landrace PHB-0683, originated in Monçao (Portugal) of the market class Cranberry, and the less resistant commercial cultivar Great Northern “Matterhorn” PMB-0220 [12,40,78], were used. Seeds of the two genotypes were sterilized by rinsing with ethanol (100%) for 30 s, followed by incubation in 5% sodium hypochlorite for 5 min, washed 5–6 folds with sterile distilled water and placed on wet sterile paper in Petri’s dishes for 72 h to them to germinate. Germinated seeds were sown in pots with a mix of artificial substrate of perlite/vermiculite (1/2 *w/w*). Each pot contained 3 plants that were inoculated at sowing with a fresh suspension of *Rhizobium leguminosarum* (ISP 14). Plants were watered every third day with the Rigaud and Puppo’s nitrogen-free nutrient solution [79]. Plants were grown in a culture chamber with 300 µE·m^−2^·s^−1^ lighting for 16 h at 26 °C and 8 h of darkness at 20 °C and 70% relative humidity for 28 days. At this time, which coincides with the end of the vegetative stage and beginning of flowering in these two genotypes, the pots were randomly separated into two groups. Nine pots each containing three plants were used for each treatment and genotype. One of the groups was subjected to 10 days of drought by withholding watering and the other was maintained with regular irrigation, as the control one. During the growth of plants, gravimetric determination of soil water content (SWC) was estimated, following the method described in previous works [12]. Finally, samples of the fourth trifoliate leaves were collected, frozen with liquid nitrogen, and stored at −80 °C for gene expression and biochemical analyses.

### 4.2. Physiological Analysis

Drought effects on the plant biomass of roots and shoots were measured in the controls, well-irrigated, and drought-stressed plants. Whole plants were collected 10 days after the beginning of the treatment and shoots and roots were weighed to obtain their fresh weight (FW). Tissue samples were desiccated at 75 °C for 72 h and weighed again to determine the dry weight (DW). Whole plant biomass was estimated as the sum of roots and shoots dry weights.

Additionally, relative leaf water content (RWC) was measured on the third trifoliate leaf as in previous work [12]. For this, after leaf sampling collection from the control and treated plants, the third trifoliate leaf from each plant was weighted and its FW was obtained. Then, the leaves were soaked with distilled water, left overnight at 4 °C, and its maximum turgor weight (TW) was obtained. Then, the same leaves were dried for 24 h at 75 °C to achieve DW and RWC obtained according to the following formula: RWC (%) = ((FW − DW)/(TW − DW)) × 100.

### 4.3. Chlorophyll Determination

Extraction of total chlorophyll from crushed leaf tissues was done following the method described by Lichtenthaler [80]. Briefly, 1.5 mL of 80% acetone was added to 90 mg of tissue. The mix was vortexed to homogenize it, centrifuged at 4 °C 3000× *g* for 10 min and the supernatant was collected in tubes kept out of direct light. This procedure was repeated three more times and the four supernatants were mixed. Afterward, the absorbance of the samples was measured at 663 and 645 nm, and the content of chlorophylls estimated according to the following formulas: Chlorophyll A (µg/mL = 12.25 (Abs_663_) − 2.79 (Abs_646_)) and chlorophyll B (µg/mL = 21.5 (Abs_646_) − 5.1 (Abs_663_)) content in leaf tissues was determined. Chlorophyll expressed as mg Chl/ g FW.

### 4.4. Determination of Catalase and Superoxide Dismutase Activities

Catalase activity was measurement by the disappearance of hydrogen peroxide at 240 nm [81]. First, the leaf extracts were obtained by homogenizing pulverized tissue with extraction buffer, which contain 100mM phosphate buffer (pH 7); 100 mM EDTA and triton X-100 (0.1%) *v*/*v*, at a relation 1:3 (*w*/*v*). The homogenized extracts were centrifuged at 20,000× *g* for 10 min at 4 °C. Then, the supernatants were collected, and low molecular weight compounds were removed by dialysis in a PD Spin Trap G-15 column (GE Healthcare) at 800 g for 1 min at 4 °C. Catalase activity was determined using 25 µL of crude extract, added to 975 µL of reaction buffer, which was composed by 50 mM phosphate buffer (pH 7) and hydrogen peroxide (0.05%), and the disappearance of hydrogen peroxide was recorded at 240 nm for 200 s. Enzyme activity unit was defined as the amount of enzyme decomposing 1.0 mole of hydrogen peroxide per minute. Calculations used an absorbance coefficient of 431 M^−1^ cm^−1^. Activity expressed as U/mg protein. Total protein content was determined according to Bradford assay [82].

Superoxide dismutase activity was measured using a commercial kit (Canvax biotech, S.L.). Leaf material was homogenized on ice using chilled extraction buffer (0,1M tris-HCl (pH 7.4); 0,5% Triton X-100, 5 mM β-ME and 0.1 mg/mL PMSF) at a relation 1:3 (*w*/*v*). Afterward, the mix was centrifuged at 20,000× *g* for 10 min at 4 °C and the supernatant was collected and dialyzed in a PD Spin Trap G-15 column (GE Healthcare) at 800× *g* for 1 min at 4 °C. SOD activity assay was carried out by measuring the disappearance of the colored tetrazolium (WST-1) formazan at 450 nm. SOD activity was estimated by the % inhibition of formazan obtained by reduction of tetrazolium salts. One-unit SOD specific activity was the amount of protein causing a 50% inhibition of formazan per mg protein. Bradford assay was used to determine protein content [82].

### 4.5. Nucleic Acid Isolation and Quantification

Total RNA purification was extracted using 50–100 mg of frozen leaf tissue. One mL of Trizol (Nzyol Tzytech) was added to this amount of tissue, homogenized by vortex and incubated for 5 min at room temperature. Next, 200 µL of chloroform were added, the mix was homogenized and incubated 8 min at room temperature. After the incubation, samples were centrifuged at 11,000× *g* at 4 °C for 10 min, and 400–600 µL of aqueous phase were collected, transferred to new tubes and 0.8 V isopropanol were added. After gentle mixing samples were incubated 5 min at RT and centrifuged at 11,000× *g* for 15 min at 4 °C to precipitate the RNA and the resulting aqueous phase was removed. Then, 1 mL of 75% ethanol was added to the RNA pellet and centrifuged 5 min at 7500× *g* at 4 °C. After this, the supernatant was carefully removed. and the RNA was resuspended in 200 µL of miliQ water to which 132 µL of lithium chloride 8M were added and incubated at 0 °C overnight for a second precipitation of RNA. The RNA samples were centrifuged at 11,000× *g* at 4 °C for 20 min. Supernatant was removed and washed with 75% ethanol before their last centrifugation at 7500× *g* at 4 °C for 5 min. Afterward, supernatant was removed, RNA pellets let dry for several minutes and resuspended in 40 µL of miliQ water. Quantification and purity of the total RNA samples were measured using a NanoDrop spectrophotometer and by visual observation after agarose gel electrophoresis.

### 4.6. Analysis of Gene Expression

First, genomic DNA was removed from the RNA samples, treatment with DNase I (New England Biolabs) at 37 °C for 10 min. Synthesis of first strand cDNA was done using 2.5 µg of DNase-treated RNA using PrimeScript™ reverse transcriptase (TAKARA) following the manufacturer’s instructions. Analysis of gene expression was carried out by qRT-PCR using iQ SYBR-Green supermix (Bio-Rad) with specific primers for each gene (Supplementary Table S2) in an iCycler iQ System (Bio-Rad). The program used was based in an initial denaturation and an activation of a Taq polymerase at 95 °C for 5 min followed of 40 cycles at 95 °C for 30 s, 60 °C for 30 s, 72 °C for 30 s and, finally, 80 cycles at 60 °C for 30 s. Relative expression of each gene for control and drought-stressed samples was normalized to that of *Actin-2* and estimated according to Livak and Schmittgen, 2001 [83]. 

### 4.7. Analysis of Promotor Regulatory Motives in WRKY Coding Genes

1.5 Kb DNA sequences of the 5′ upstream regions of each gene were obtained from *Phytozome* database v12 (https://phytozome.jgi.doe.gov/) [84], and search for regulatory *cis*-elements was done using plant CARE software (http://bioinformatics.psb.ugent.be/webtools/plantcare/html/) [85].

### 4.8. Experimental Design and Statistical Analysis of Data

At least three independent samples were analyzed in this research for each condition (Control and drought) and genotype (PHB-0683 and PHA-0246). Each sample consisted in three pots per condition, each containing three plants. Statistical analysis was done by Student’s *t*-test and ANOVA using GraphPad Prism 6 software package (https://www.graphpad.com/).

## 5. Conclusions

**Figure 5:**
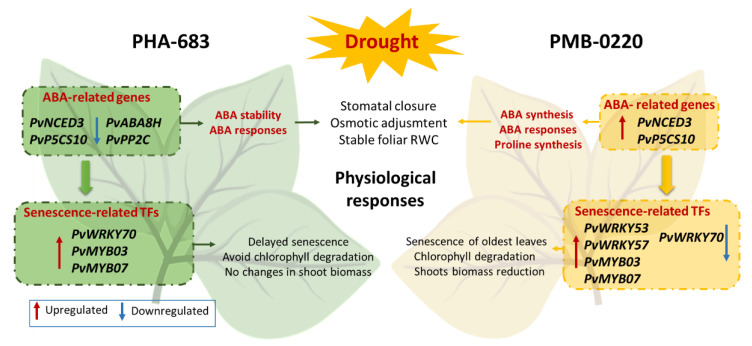
Schematic representation of gene expression patterns of key transcription factors related to senescence and drought responses in leaves from control and 10 days-drought-stressed PMB-0220 and PHA-0683 plants. Only key genes whose expression levels appeared as upregulated or downregulated with respect to control well-irrigated plants are shown.

Results presented here indicate that the resistance to drought in PMB-0220 and PHA-0683 common bean accessions is regulated by distinct molecular mechanisms. Differential regulation of ABA synthesis and ABA signaling related genes among the two genotypes and the control of the drought-induced senescence have a relevant contribution to the higher resistance level of PHA-0683 accession (Figure 5). 

## Figures and Tables

**Table 1:** Concentration of chlorophyll a, b and total chlorophyll in the 2nd, 3rd, 4th and 5th trifoliate leaves of *P. vulgaris* PMB-0220 and PHA-0683 subjected to well-watered and water deficit conditions for 10 days.

		[Chlorophyll a]		[Chlorophyll b]		[Chlorophyll a + b]	
		PMB-0220	PHA-0683	PMB-0220	PMB-0683	PMB-0220	PHA-0683
**2nd TFL**	**Control**	1.270 ± 0.209	1.475 ± 0.279	0.634 ± 0.115	0.732 ± 0.151	2.013 ± 0.336	2.362 ± 0.417
	**Drought**	0.501 ± 0.144	1.485 ± 0.201	0.429 ± 0.207	0.634 ± 0.070	1.078 ± 0.451	2.329 ± 0.294
**3rd TFL**	**Control**	1.664 ± 0.225	1.673 ± 0.160	0.789 ± 0.146	0.873 ± 0.148	2.880 ± 0.085	2.795 ± 0.317
	**Drought**	1.452 ± 0.195	1.589 ± 0.186	0.704 ± 0.071	0.729 ± 0.044	2.246 ± 0.140	2.546 ± 0.230
**4rd TFL**	**Control**	1.599 ± 0.215	1.669 ± 0.172	0.891 ± 0.082	0.877 ± 0.125	2.867 ± 0.142	2.798 ± 0.267
	**Drought**	1.546 ± 0.251	1.657 ± 0.148	0.778 ± 0.118	0.906 ± 0.148	2.551 ± 0.294	2.813 ± 0.267
**5th TFL**	**Control**	1.587 ± 0.178	1.797 ± 0.174	0.687 ± 0.156	0.886 ± 0.125	2.685 ± 0.166	2.950 ± 0.251
	**Drought**	1.628 ± 0.134	1.776 ± 0.160	0.779 ± 0.191	0.889 ± 0.297	2.644 ± 0.206	2.926 ± 0.487

## Supplementary Materials

The following are available online at https://www.mdpi.com/2223-7747/9/12/1815/s1, Table S1: Cis regulatory motifs found in the promoter regions of selected senescence related WRKY genes; Table S2: List of primers used in this study. Figure S1: Superoxide dismutase (SOD) (A) and catalase (B) enzymatic activity in leaves from PMB-0220 and PHA-0638 common bean from control and 10 days water-stressed plants.
